# Mapping of protein phosphatase-6 association with its SAPS domain regulatory subunit using a model of helical repeats

**DOI:** 10.1186/1471-2091-10-24

**Published:** 2009-10-16

**Authors:** Julien Guergnon, Urszula Derewenda, Jessica R Edelson, David L Brautigan

**Affiliations:** 1 Center for Cell Signalling, University of Virginia School of Medicine, Charlottesville, VA, 22908 USA; 2 Department of Microbiology, University of Virginia School of Medicine, Charlottesville, VA, 22908 USA; 3 Department of Molecular Physiology & Biological Physics, University of Virginia School of Medicine, Charlottesville, VA, 22908 USA

## Abstract

**Background:**

Helical repeat motifs are common among regulatory subunits for type-1 and type-2A protein Ser/Thr phosphatases. Yeast Sit4 is a distinctive type-2A phosphatase that has dedicated regulatory subunits named Sit4-Associated Proteins (SAPS). These subunits are conserved, and three human SAPS-related proteins are known to associate with PP6 phosphatase, the Sit4 human homologue.

**Results:**

Here we show that endogenous SAPS subunit PP6R3 co-precipitates half of PP6 in cell extracts, and the SAPS region of PP6R3 is sufficient for binding PP6. The SAPS domain of recombinant GST-PP6R3 is relatively resistant to trypsin despite having many K and R residues, and the purified SAPS domain (residues 1-513) has a circular dichroic spectrum indicative of mostly alpha helical structure. We used sequence alignments and 3D-jury methods to develop alternative models for the SAPS domain, based on available structures of other helical repeat proteins. The models were used to select sites for charge-reversal substitutions in the SAPS domain of PP6R3 that were tested by co-precipitation of endogenous PP6c with FLAG-tagged PP6R3 from mammalian cells. Mutations that reduced binding with PP6 suggest that SAPS adopts a helical repeat similar to the structure of p115 golgin, but distinct from the PP2A-A subunit. These mutations did not cause perturbations in overall PP6R3 conformation, evidenced by no change in kinetics or preferential cleavage by chymotrypsin.

**Conclusion:**

The conserved SAPS domain in PP6R3 forms helical repeats similar to those in golgin p115 and negatively charged residues in interhelical loops are used to associate specifically with PP6. The results advance understanding of how distinctive helical repeat subunits uniquely distribute and differentially regulate closely related Ser/Thr phosphatases.

## Background

Helical repeat motifs such as ANK, HEAT, and ARM are thought to primarily mediate protein-protein interactions (see reviews[1-3]). Helical repeat motifs are a recurrent theme among regulatory subunits for different protein Ser/Thr phosphatases. Best studied is the A or PR65 subunit of PP2A, an all-helical subunit first designated to consist of Armadillo (ARM) sequence repeats, that were later called HEAT repeats [4], a name derived from proteins with related sequence motifs: Huntingtin's, elongation factor, A subunit of PP2A and TOR. The 3D structure of the A subunit of PP2A alone [5], as a dimer bound to the PP2A catalytic subunit [6], and as a scaffold to assemble PP2A heterotrimers [7-9], showed the all-helical organization and revealed differences in overall conformation due to association with the other subunits. The extended arc of helices is shaped like a banana in the monomer or heterodimer and closes to a horseshoe-shaped conformation in the heterotrimer. In addition, in the ABC trimers the regulatory B'56 subunit of PP2A was found to be a HEAT-like helical repeat protein that contacts both the A and C subunits. The structure of B'56 was unexpected because it was not predicted based on sequence alignments with other HEAT-repeat proteins. Another example of helical repeat motifs in protein phosphatase subunits is the MYPT1 subunit for PP1, with 8 ankyrin repeats [10]. In the 3D structure these repeats form an arc of alpha helices to engage the top surface of the PP1 catalytic subunit and enwrap the C-terminal tail that protrudes from the top surface of the subunit. Both the ANK repeats as well as a separate structural element consisting of an alpha helix plus a neighboring strand with the canonical RVxF motif make contacts with the PP1 catalytic subunit. Based on these examples there is the expectation that other phosphatase regulatory subunits might be comprised of helical repeat structures and use these repeats to mediate subunit-subunit association.

The yeast Sit4 phosphatase is related in sequence and properties to members of the type-2A family of protein Ser/Thr phosphatases [11]. Strains with temperature-sensitive mutations (*sit4*^*ts*^) are rescued by ectopic expression of human PP6 [12], but not the close relative PP4, showing functional complementation across species, but specificity for the individual type of catalytic subunit. The results argue for distinct lines of evolutionary descent for PP2A, PP6 and PP4, with a high degree of conservation within each line. Yeast Sit4 has multiple associated subunits that co-immunoprecipitate, first named Sit4-Associated Proteins (SAP) [13]. Sequence alignments using a region common in the yeast SAP identified SAPS in various species, including three human proteins (KIAA1115, KIAA0685 and C11orf23), which were renamed PP6R1, PP6R2 and PP6R3 and shown to co-precipitate with PP6, but neither PP4 nor PP2A [14]. The sequence motif in yeast and human proteins, as well as in other species, has been designated as a "SAPS" domain by PFAM . These SAPS domain proteins are proposed to function as specific regulatory subunits for PP6. Truncation of the C-terminal region of PP6R1 did not compromise co-precipitation with PP6, showing that the designated SAPS domain was sufficient for binding the catalytic subunit.

The physiological function(s) of this family of SAPS domain proteins as specific regulatory subunits is not yet well understood. Knockdown of individual SAPS domain subunits by siRNA mimics knockdown of PP6 catalytic subunit itself in terms of effects on putative individual substrates such as IκBε and DNA-PK [14,15]. The results argue that all three SAPS subunits can individually associate with PP6, but the different PP6 complexes have non-overlapping substrate specificity. The conservation of SAPS function in PP6R1, PP6R2 and PP6R3 subunits is demonstrated by their ability to co-precipitate endogenous Sit4 when expressed in yeast strains deleted for their endogenous SAPs [16]. Furthermore, the human SAPS proteins partially restored functions in these strains. Proteomics discovered association of PP6R1 with multiple ankyrin repeat domain proteins (Ankrd), suggesting a heterotrimeric organization of PP6 [17]. This makes the mammalian PP6 different from the yeast Sit4 because the Ankrd genes are not found in yeast and there is no evidence for Sit4 heterotrimers. This opens the possibility that the Ankrd subunits function to target and regulate PP6 actions in addition to the SAPS subunits. Here we analyzed the properties of the PP6R3 subunit using partial proteolysis, deletions and co-precipitation assays from cells. The SAPS domain sequences were used to produce alternate structural models of helical repeat motifs, and these models were used to select sites for point mutations that were assayed by co-precipitation. The results indicate the SAPS domain subunits of PP6 use a newly-found variation of the helical repeat theme to achieve selective recognition of PP6c.

## Methods

### Antibodies, Immunoprecipitation and immunoblotting

Anti-FLAG antibodies were purchased from Sigma-Aldrich and used at a dilution of 1:5000 (Rabbit) or 1:3000 (mouse). Goat anti-rabbit Alexa Fluor 680 and donkey anti-sheep Alexa Fluor 680 were purchased from Molecular Probes and Invitrogen and used at a 1:5000 dilution. Goat anti-mouse IRDye 800 and anti-chicken IRDye 800 antibodies were purchased from Rockland Immunochemicals and used at a 1:5000 dilution. Rabbits were immunized with purified recombinant GST made in bacteria, and anti-GST antibodies were affinity-purified from serum and used at a 1:5000 dilution. Antibody against PP6 [14] was used at a 1:3000 dilution. Sheep were immunized with purified recombinant GST-PP6R3 made in bacteria, and antibodies were affinity-purified from serum against MBP-PP6R3 and used at a 1:2000 dilution. Anti-PP6R3 beads were prepared as follows: 200 μl of protein-G agarose beads were incubated with 400 μg of affinity purified anti-PP6R3 in 2 ml at room temperature for 1 h. Beads were then washed twice by centrifugation with 1 ml of 0.2 M sodium borate solution, pH 9.0. Beads were resuspended in 1 ml of sodium borate and a final concentration of 20 mM disuccinimdyl pimelimate added. After incubation at room temperature for 30 min the coupling reaction was stopped by washing the beads once by centrifugation, with resuspension and incubation in 0.2 M ethanolamine for 2 h at room temperature. Beads were washed twice by centrifugation and resuspended in PBS. Chicken anti-PP6R3 IgY antibodies were prepared using GST-PP6R3 as immunogen by Aves Laboratories (California).

For immunoprecipitation cells were washed with ice-cold PBS then lysed with 20 mM Tris-HCl, pH 7.5, 150 mM NaCl, 1% NP-40, 5 mM EDTA, 5 mM NaF and protease inhibitor mixture set V, EDTA-free (Calbiochem). After 15 min on ice cells were scraped and the suspension transferred to a tube, mixed on a vortex, and centrifuged at 20,000 × g for 20 min. Supernatants were used for immunoprecipitation with 30 μl of immobilized anti-PP6R3 or anti-FLAG beads (M2, Sigma) overnight at 4°C with gentle rotation. Beads were washed 3 times with the lysis buffer then boiled 5 min in 2× SDS sample buffer. SDS-PAGE was done using acrylamide/Bis 29:1 as previously described [14] or using pre-cast CRITERION gradient gels (BioRad). Gels were stained with Coomassie blue or GelCode (Thermo Scientific), or proteins were transferred by electrophoresis onto nitrocellulose for immunoblotting, and developed using LI-COR Odyssey Infrared Imaging System (LI-COR biotechnology). This scanner provides quantitative analysis with a extended range of linear response.

### Plasmids and PCR

DNA of human PP6R3 was amplified by PCR from HeLa cDNA generated by Thermoscript poly(dT) reverse transcription-PCR (Invitrogen) following the manufacturer's protocol. The primers for PCR of PP6R3 were 5'-GAA TTC ATG TTT TGG AAA TTT GAT CTT C-3' as the forward primer and 5'-CTC GAG CAC TTC AGT GAA TGG CCC TGT ATC ACT G-3' as the reverse primer. PP6R3 fragments 1-355 and 1-513 were generated using the same forward primer as PP6R3 full length, and 5'-CTCGAGTCAAAGCAGGCTGGATATCAACCTAATG-3' and 5'-CTCGAGTCAGTTCCTCTTGTTAGTTTCTCCTAAG-3' as reverse primers respectively. PP6R3 512-873 was generated using 5'-GAATTCACGGTAGATCTAATGCAAC-3' as the forward primer and 5'-CTCGAGTCATACAGGGCCATTCACTGAAGTG-3' as the reverse primer. For directed mutagenesis, the QuickChange site-directed mutagenesis kit was used according to manufacturer's protocol. Primers for E63-E64 to K were 5'-GTCTCATTCATTATAAAAAAACCACCTCAAGACATGGATG-3' as the forward primer and 5'-CATCCATGACTTGAGGTGGTTTTTTTATAATGAATGAGAC-3' as the reverse primer. Primers for mutation of D113 to R were 5'-GCTTCCTCCTAAACCGTTCCCCTTTGAATCCACTAC-3' and 5'-GTAGTGGATTCAAAGGGGAACGGTTTAGGAGGAAGC-3' as forward and reverse primers respectively. Primers to mutate E204-E205 to K were 5'-GTTCATCCATCGCAAAAAAAAGATCGACATTCAAATGC-3' as the forward primer and 5'-GCATTTGAATGTCGATCTTTTTTTTGCGATGGATGAAC-3' as the reverse primer. Primers for mutation of E259-E262 to K were 5'-CAAATATTTTCCACAAGAAGAAAAATAAGTCAGCCATAGTCAG-3' and 5'-CTGACTATGGCTGACTTATTTTTCTTCTTGTGGAAAATATTTG-3' as forward and reverse primers respectively.

### Recombinant Protein Production and Analyses

Full length PP6R3 (accession Q5H9R7) was subcloned in pGEX-4T-1 vector for production of recombinant GST-PP6R3 in BL-21 strain of E. coli that was purified using glutathione Sepharose following manufacturer's instructions. PP6R3 SAPS domain (residues 1-513) was subcloned by PCR and ligated into pET30 vector, expressed in BL21 cells, and purified by metal ion affinity chromatography using Ni-NTA Agarose (Qiagen).

Circular dichroic (CD) spectrum of purified recombinant PP6R3(1-513) at 0.2 mg/ml in Tris HCl pH 7.4 with 0.15 M NaCl was recorded with a Aviv model 215 spectropolarimeter.

Purified GST-PP6R3 (2 mcg) was incubated with 10 ng of TPCK-trypsin in 20 mM Tris pH 7.5 at room temperature for times ranging from 0 to 10 minutes. Reaction was stopped by transferring aliquots of the reaction into ice-cold denaturing buffer (final concentrations 2% SDS, 50 mM Tris pH 7.5, in 10% glycerol). Samples were analyzed by SDS-PAGE and immunoblotting. Molecular size standards were BioRad Precision Plus.

### Cell culture and transfections

HEK293, HEK293T and HepG2 cells were grown in MEM medium with 2 mM L-glutamine and 10% FBS. HeLa cells were grown in DMEM medium containing 10% FBS. Jurkat cells were grown in RPMI medium with 2 mM L-glutamine and 10% FBS. Cells were grown at 37°C in humidified 5% CO_2 _atmosphere. HEK293 cells at 40-50% confluence in 10 cm dishes were transiently transfected using 4-10 μg of plasmid DNA mixed with Arrest-in (Open Biosystems) for 20 min at room temperature before addition to cells. Cells were harvested after 24 h and extracts prepared for immunoprecipitation.

### Sequence Alignments and Structure modelling

Amino acid sequences of PP6R3 were not recognised as helical repeat motifs by standard algorithms (including by PFAM, which was used to define the SAPS family). The PP6R3 amino acid sequence did not align with signature sequence motifs for either the ARM or HEAT helical repeats. Furthermore, the human PP6R3 sequence did not align with any other protein of known 3D structure, when using standard algorithms. We used the ALSCRIPT program [18] to align sequences as shown in Figure four.

Modelling of the SAPS protein was carried out using program REP [17]. REP was designed to identify structural repeats from protein sequences. The program has been trained on profiles from several known ARM and HEAT repeat proteins, but did not identify repeat motifs in the PP6R3 sequence when used with standard thresholds. REP in a low-confidence, no-thresholds-applied mode detected sequence repeats in PP6R3, but failed to assign them as ARM, HEAT or ANK repeats.

As an alternative we opted to use the 3D-Jury protein structure prediction Meta Server . The metaserver first generates 3D models using diverse structure prediction methods, and then compares and scores models, based on their consistency [19]. This approach allowed us to generate possible models for the SAPS domain, based on other proteins that contain either ARM or HEAT repeats. These models were depicted as structures in Figure five using PyMOL .

### Chymotrypsin digestion of wild-type vs. mutated FLAG-PP6R3

Human 293T cells at <40% confluence in 100 mm dishes were transfected using 30 μl Arrest-In (Open Biosystems) diluted in 50 μl Opti-MEM with 5 μg of plasmid DNA encoding either wild type PP6R3 or quadruple mutant PP6R3 (E204K, E205K, E259K, E262K). After 24 h, cells were lysed using 1% NP-40 buffer and centrifuged at 13,000 rpm for 15 min. Aliquots of 500 μl of the supernatant were incubated with 50 μl M2 Agarose beads (Sigma, A2220) overnight at 4°C with rocking. Beads were washed 3× with 100 μl of wash buffer A (1% NP-40 plus 2 mM ATP and 5 mM MgCl_2_), two times with 100 μl wash buffer B (50 mM Tris pH 8.0, 500 mM NaCl), and one time with 100 μl wash buffer C (50 mM Tris pH 8.0, 150 mM NaCl). The beads were resuspended in 120 μl wash buffer C and 20 μl removed as time zero control. Chymotrypsin was added to a final concentration of 0.5 ng/μl. At various times 20 μl aliquots were removed, mixed with 20 μl 2× SDS buffer and heated to 100°C for 5 min.

Samples were resolved by 4-15% gradient SDS-PAGE, transferred onto nitrocellulose by a semi-dry protocol, and the filter blocked with 5% non-fat milk in Tris-buffered saline plus 1% Tween 20. The filters were probed with chicken anti-R3 antibodies (1:2000) or anti-FLAG antibodies (Sigma F7425, 1:1000), developed with fluorescent secondary antibodies, and scanned 2D images were captured by an Odyssey 2D infrared scanner (LiCor Industries). As a control a duplicate sample of washed beads resuspended in 120 μl wash buffer C was incubated at 100°C for 5 min. Following centrifugation the supernatant with denatured GST-PP6R3 was incubated with chymotrypsin, and samples at various time points processed as described above. Molecular size standards were BioRad Precision Plus.

## Results

**Figure 1:**
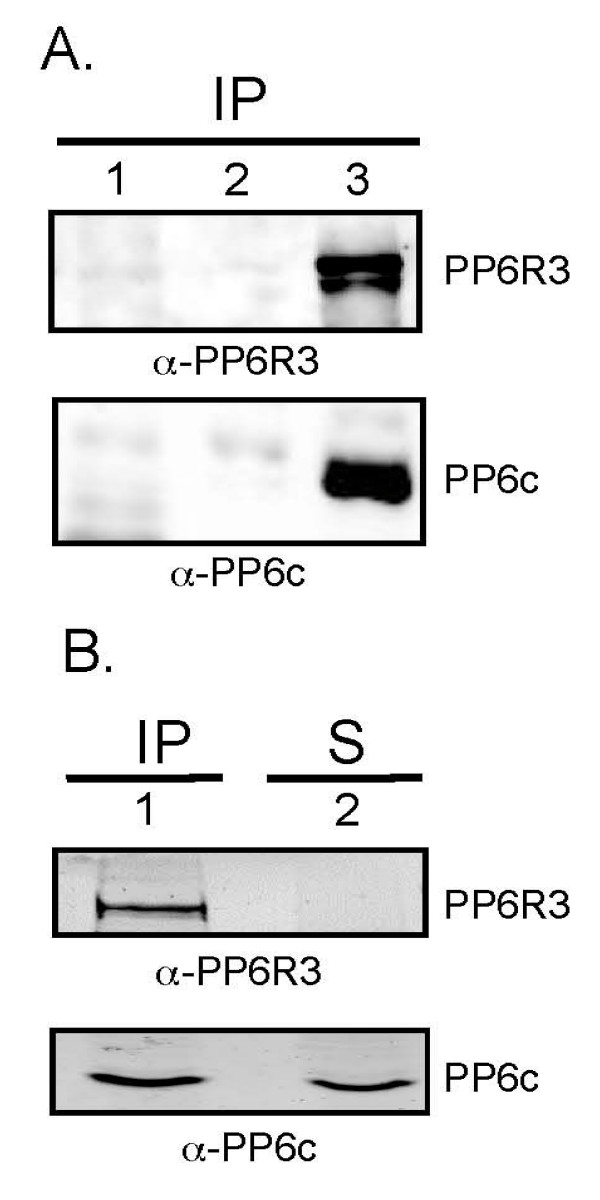
**Co-immunoprecipitation of endogenous PP6R3 and PP6c**. (A) Immunoprecipitates (IP) were prepared from HeLa cells with pre-immune sheep serum (lane 1), blank beads (lane 2) or anti-PP6R3 sheep antibody (lane 3). Precipitates were analyzed by immunoblotting for PP6R3 (upper panel) and PP6c (lower panel). (B) Co-immunoprecipitation of PP6R3 and PP6c from HepG2 cells with anti-PP6R3 antibody (lane 1). The PP6R3 and PP6c remaining unbound were detected by immunoblotting the supernatant (S, lane 2) for PP6R3 (upper panel) and PP6c (lower panel)

The SAPS domain appears in three human proteins, based on a common region of sequence identity and similarity (14). We prepared individual antibodies to each and found that PP6R3 (*a.k.a*. SAPS3) was most efficiently immunoprecipitated from HEK293 cell extracts (Figure 1). PP6R3 co-precipitated endogenous PP6 catalytic subunit (PP6c) that was detected by immunoblotting (Figure 1A, lane 3). Immunoprecipitation was specific because neither PP6c nor PP6R3 was in control samples prepared using blank beads or non-immune primary antibody (Figure 1A, lanes 1, 2). After two rounds of immunoprecipitation the extracts were fully depleted of PP6R3 (Figure 1B, lane 2) as well as 50% of the endogenous PP6c. The other 50% of PP6c in the supernatant presumably was associated with other subunits besides PP6R3. The same results were obtained with co-precipitation from extracts of HeLa, Jurkat and HepG2 cells (not shown). We concluded that endogenous PP6R3 stably associates with about half of the total PP6c in tissue culture cell lines, making it the major partner for PP6 and a suitable candidate for further study as representative of the SAPS domain subunits.

### Trypsin digestion and CD Spectrum of recombinant PP6R3

**Figure 2:**
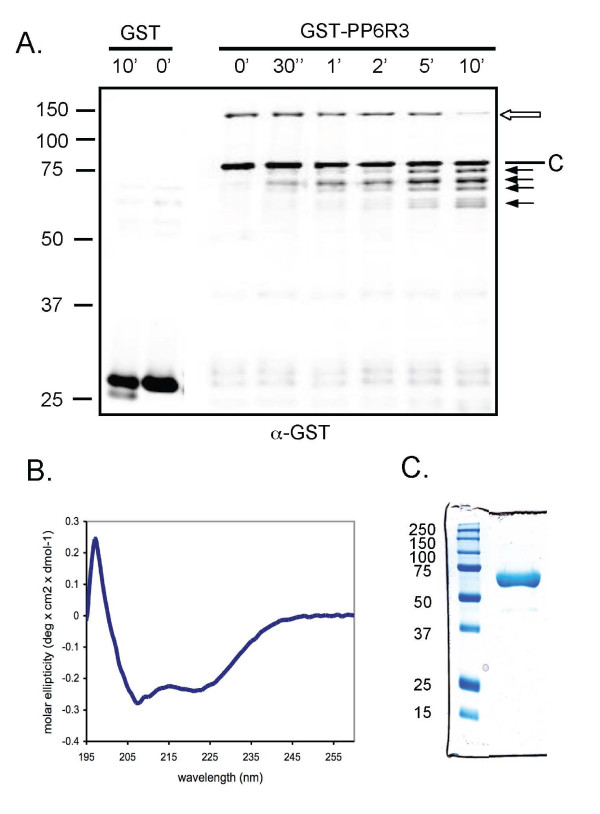
**Trypsin fragmentation of GST-PP6R3 and CD spectrum of SAPS domain**. (A) Recombinant GST-PP6R3 (upper band, open arrow) was digested with trypsin from 0 to 10 min. The GST-PP6R3 and fragments were detected using an anti-GST antibody and a non-specific band (labelled C) that was not digested served as loading control Trypsin caused time-dependent formation of four digestion products (arrows). GST was digested under identical conditions for 10 min (left 2 lanes). The migration of molecular size standards is indicated to the left side of the frame. (B) Circular dichroic (CD) spectrum of purified recombinant PP6R3(1-513) plotted as molar ellipticity (× 10-5) vs. wavelength in nm. (C) Recombinant His6 tagged SAPS domain residues 1-513, purified and stained with Coomassie after SDS-PAGE. Left lane, size standards in kDa; right lane is protein used for CD spectrum.

We expressed recombinant GST-PP6R3 fusion protein in bacteria, affinity-purified the protein and subjected it to partial proteolysis by trypsin to examine its fragmentation (Figure 2A). The purified fusion protein migrated as a single band at 140 kDa and, as expected, was reactive with both anti-GST and anti-PP6R3 antibodies. The actual molecular mass of PP6R3 is 97.7 kDa (873 residues), so the fusion protein migrates much less (at a higher Mr) than predicted. Another protein at ~80 kDa was detected by anti-GST immunoblotting, but not by anti-PP6R3 blotting, and this protein was not digested by trypsin under the conditions used, therefore we used it as a convenient loading control for aliquots taken at different time points. Multiple trials were conducted to optimize conditions for time-dependent conversion of the GST-PP6R3 into fragments. Digestion with trypsin at 1/5000 (w/w) at room temperature and pH 7.5 (shown in Figure 2) gave progressive loss of the full length GST-PP6R3 fusion protein that was nearly complete by 10 min. Treatment of GST with trypsin under identical conditions showed that GST was relatively resistant to digestion (Figure 2A, left 2 lanes). Therefore, we could use anti-GST immunoblotting to track cleavage of GST-PP6R3. The full-length fusion protein was converted into four major fragments that were recognised by anti-GST (Figure 2A) as well as with anti-PP6R3 (not shown). One fragment formed first, as early as 30 sec, and remained as the primary product after 10 min. All the major products were >60 kDa, suggesting that trypsin cleaved following the GST fused to the N terminus at a point ca. 330-350 residues into the PP6R3 sequence. We concluded that the N-terminal region of PP6R3 up to that point has a conformation in solution that limits digestion by trypsin, even though there are many lysine and arginine residues in this region.

In addition, a recombinant His6-tagged PP6R3 protein, residues 1-513, was purified from bacteria. The CD spectrum was recorded (Figure 2B) and the purity of the protein verified by Coomassie staining after SDS-PAGE (Figure 2C). The CD spectrum with minima at 208 and 222 nm is characteristic of proteins that are predominantly made of alpha helices [20]. Together these results suggest that the SAPS domain is composed of alpha helical secondary structures in a conformation that limits trypsin digestion.

### Mapping PP6 binding to SAPS domain in PP6R3

**Figure 3:**
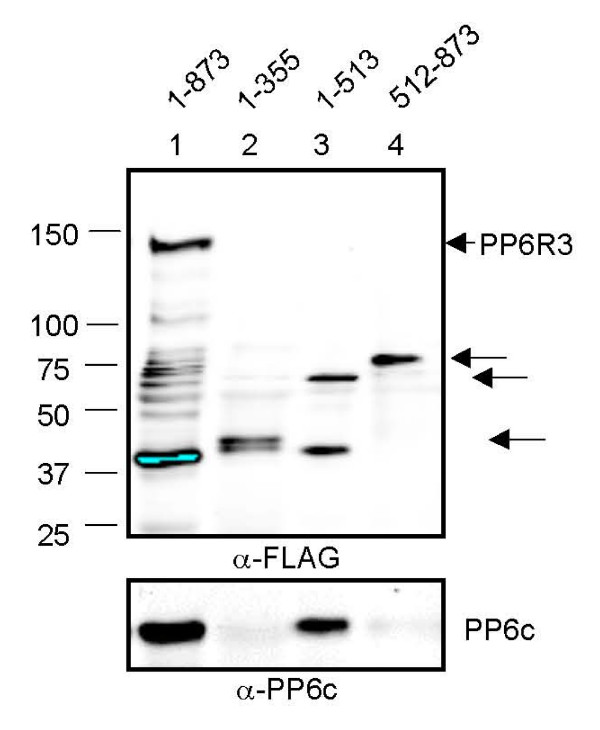
**Deletion mapping of PP6c binding to PP6R3**. FLAG-tagged full length PP6R3 (lane 1), or residues 1-355 (lane 2), 1-513 (lane 3) or 512-873 (lane 4) were expressed in HEK293 cells and immunoprecipitated with anti-FLAG antibody. FLAG-tagged proteins (upper panel) and co-immunoprecipitated endogenous PP6c (lower panel) were detected by immunoblotting.

To map the region of PP6R3 required for PP6c binding we expressed the following in HEK293 cells: 1) FLAG-tagged PP6R3 (full-length, residues 1-873), 2) a SAPS region (residues 1-513), 3) a C-terminal region (512-873) and 4) a truncated SAPS region (1-355) corresponding approximately to the trypsin-resistant N terminus. Cell extracts and FLAG immunoprecipitates were prepared and immunoblotted for FLAG and endogenous PP6c (Figure 3). Full length PP6R3 effectively co-precipitated PP6c (lane 1), as did the SAPS region (residues 1-513; lane 3). Less PP6c was recovered with 1-513 compared to full-length, but we attributed this to lower expression level of the 1-513 protein. The FLAG immunoprecipitate in lane 3 had about equal amounts of a protein corresponding to residues 1-513 and a smaller FLAG-tagged protein fragment. This smaller fragment is not expected to bind PP6c because of the lack of co-immunoprecipitation with the 1-355 protein of about the same size in the immunoblot (lane 2). We noted that endogenous proteolysis generated nearly the same-sized fragments (about 340 residues of PP6R3) in lanes 1, 2 and 3. PP6c did not co-precipitate at all with a C-terminal region of PP6R3 (residues 512-873) (Figure 3, lane 4). Previous data with a different SAPS subunit, PP6R1, showed residues 1-465 were sufficient for co-precipitation of PP6c, whereas residues 462-825 did not co-precipitate PP6c [14]. These new results show that in PP6R3 a region of residues 1-355 was not sufficient, but residues 1-513 could co-precipitate endogenous PP6c.

### Prediction of 3D organization of the SAPS domain

**Figure 4:**
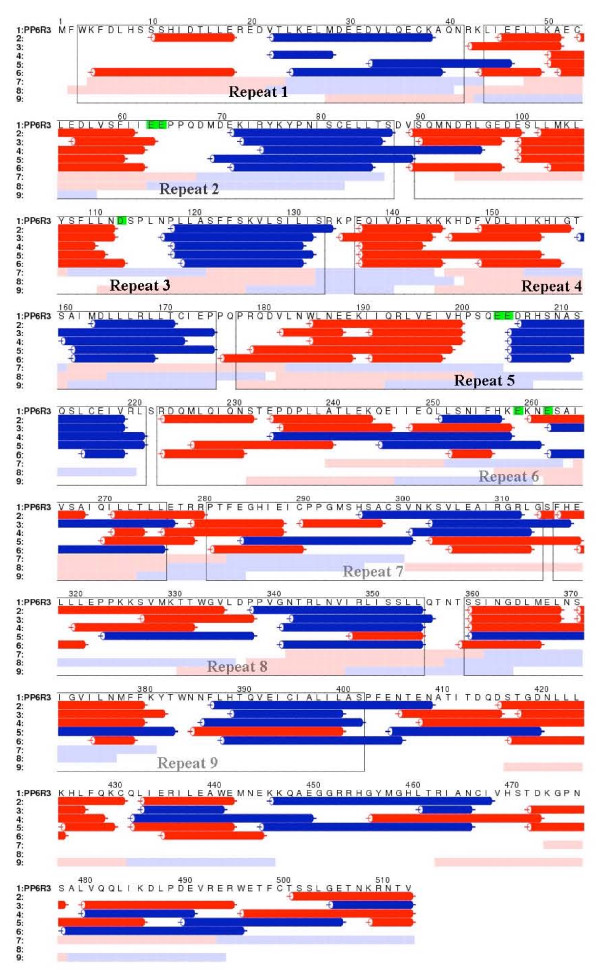
**Alternative models for helical repeats in the SAPS domain of PP6R3**. Line 1. PP6R3 primary sequence, residues 1-513. Positions of the mutations tested in Fig. 6 are highlighted in green. Lines 2-6. 3D-jury models based on: line 2- importin α; line 3- β-catenin; line 4- importin β; line 5- p115. Convex surface helices are red and concave helices are blue cylinders. Lines 7-9 are REP low confidence predictions of: line 7- HEAT; line 8- ARM; line 9- ANK repeats. For consistency with the models we have highlighted these areas pink for convex and light blue concave surfaces. Proposed helical repeat regions are boxed and labelled 1-9. We are less confident in repeats 6-9, labelled grey, compared to repeats 1-5 labeled in black.

**Figure 5:**
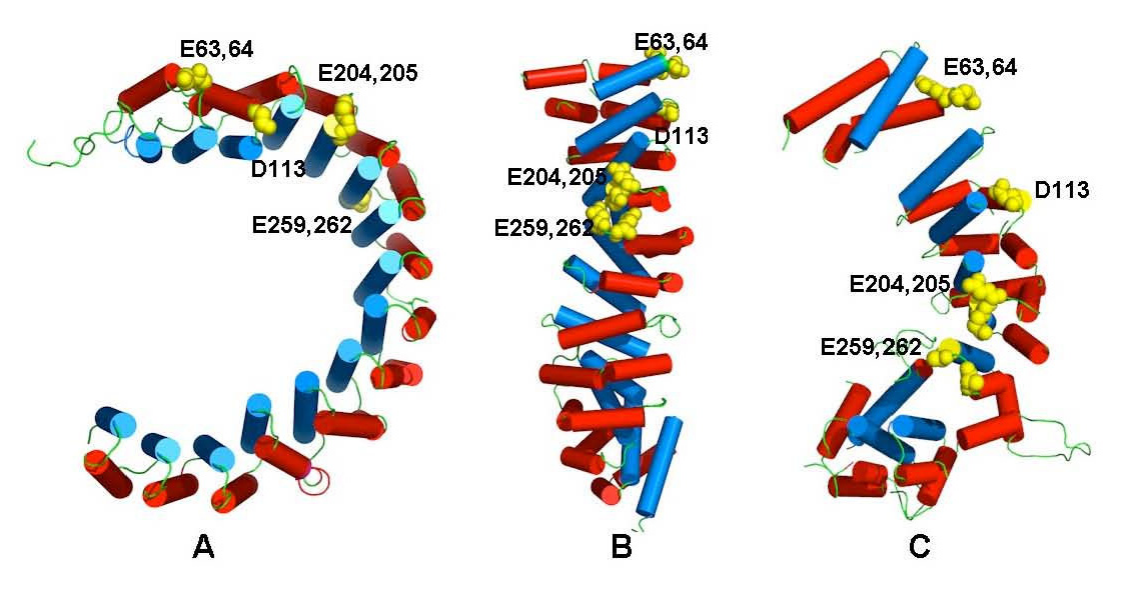
**Structural models for SAPS domain in PP6R3**. The sequence of PP6R3 residues 1-513 was used to produce alternate models based on known helical-repeat proteins (left to right): A- PP2A A subunit, B- beta-catenin, C- p115 golgin. Predicted alpha helices are shown in red (convex) and blue (concave), with intervening loops as strands. Side chains of E63, E64, D113, E204, E205, E259 and E262 are shown as space-filling models in yellow.

Alternative models for the PP6R3 SAPS region were generated by sequence alignments (Figure 4) and structural modeling by the 3D-Jury Meta Server, using proteins containing either ARM or HEAT repeats. The modelling fits regions of sequence into putative alpha helices and predicts surface loops between the helices. The highest score models (Figure 5) were based on: 1) importin alpha ARM repeat structures (PDB ID 2C1T, 1IAL, 1Q1S) (line 2, Figure 4), and 2) beta-catenin ARM domain, (1I7W, 1JDH) (line 3 Figure 4). Models based on HEAT repeats in beta importin (2BKU) and the PP2A PR65 subunit (1B3U) had lower scores, but were included as possible alternatives in Figure 4 (line 4 and 5). An unusual, new ARM repeat structure of a vesicular transport factor called golgin p115 was published recently [21]. This structure offers another alternative for modelling of PP6R3. In golgin p115, helical repeats 7-9 contain extended loops between helix 1 and 2, not previously seen in other ARM domains. This novel structure raises the possibility that residues ~223-276 in PP6R3 form a single elongated repeat, ~15 residues longer than standard (repeat 6, Figure 4).

The structure of a HEAT repeat is similar to the structure of an ARM repeat, despite the fact that the ARM motifs consist of three helices, while HEAT motifs have only two. In both cases, the repeats are stacked together to form a super-helix (or solenoid) that in the case of PP2A PR65 and importin are curved (Figure 5A), while the structures of importin or beta-catenin are elongated, with less curvature (Figure 5B). The helices in the model based on the golgin p115 structure are less uniform and their arrangement is less compact (Figure 5C). There are predicted surface loops in the golgin p115-based structure that do not appear in the other models.

We expected that the SAPS predicted structure would resemble the HEAT helical repeats in the PP2A PR65 subunit, because, after all, this protein fold binds the PP2A catalytic subunit that is nearly identical to PP6. However, this model had the lowest 3D-Jury score. The models in both Figures 4 and 5 agree in assignment of the boundaries of the helical motifs in the N terminal residues 1-230, and therefore we were confident in the phasing of these intra-repeat loops. The assignments of helices and loops past residue 230 was more difficult and the models diverged in these segments. We speculated that the interactions between PP6R3 and the PP6 catalytic subunit could be mediated by the intra-motif loops and not the inter-motif loops. We used site-directed mutagenesis to generate charge reversal mutations in acidic residues that were predicted from the modelling to be in the loops. This was to test whether these sites were required for binding PP6c and therefore allow us to discriminate between the models in Figure 5.

### Mutation analysis of the PP6R3 binding to PP6

**Figure 6:**
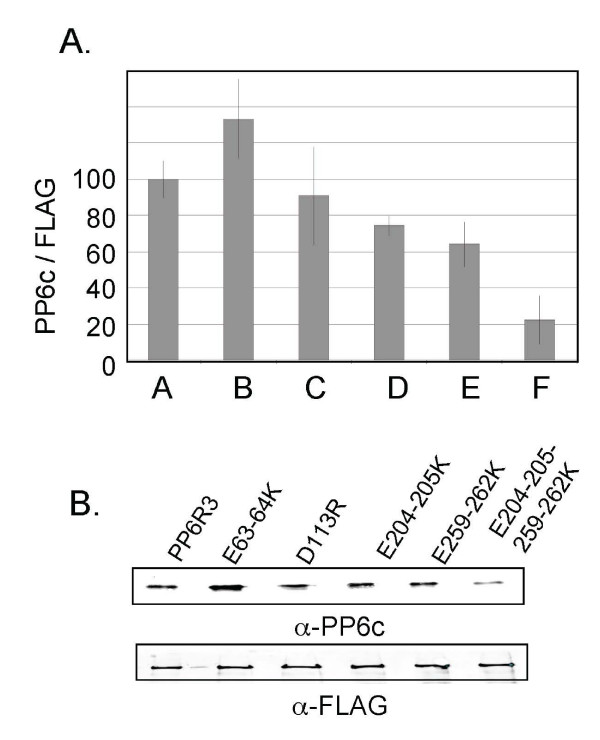
**Mapping PP6c binding by charge-reversal mutations in PP6R3**. (A) FLAG-tagged full length (FL) PP6R3 (A), and mutants E63-64K (B), D113R (C), E204-205K (D), E259-262K (E) and E204-205-259-262K (F) were expressed in HEK293 cells and immunoprecipitated using immobilized anti-FLAG antibody. Co-precipitated PP6c was quantified by fluorescent immunoblotting and normalized for the amount of FLAG-tagged protein. Results were replicated in 3 independent experiments and plotted as mean +/- SD. (B) Immunoblot of co-precipitated endogenous PP6c from one experiment (upper panel) and the FLAG-tagged PP6R3 proteins (lower panel).

We used transient expression of FLAG-PP6R3 and co-precipitation to assay binding to endogenous PP6c in cells (Figure 6). Wild type FLAG-PP6R3 and the following mutants 1) E63,64K; 2) D113K; 3) E204,205K and 4) E259,262K were expressed in HEK293 cells, and the amount of FLAG protein and co-precipitated PP6c determined by immunoblotting. The recovery of the FLAG-tagged PP6R3 proteins was nearly identical (Figure 6B) and the intensity of anti-FLAG staining was used to normalize the anti-PP6c staining, with the amount of PP6c bound to wild type PP6R3, set as 100 (Figure 6A). Multiple independent experiments gave results that were analyzed together. Dual mutation of E63 and E64 to K increased recovery of PP6c by about 30%. In contrast, other negative to positive charge mutations reduced binding of PP6c. The decrease in PP6c binding due to the D113R mutation was not significant. Dual mutations of either E204-E205 or E259-E262 produced statistically significant 25 to 35% reduction in PP6c binding. Combination of the dual mutations of 204/205 plus 259/262 resulted in the largest effect compared to wild type, an overall 80% reduction in co-precipitation of endogenous PP6c. These data indicated that the negatively charged residues in the predicted loops at residues 204, 205, 259 and 262 in PP6R3 were likely involved in association with PP6c.

### Mutations Reduce PP6c Binding without Change in Cleavage by Chymotrypsin

**Figure 7:**
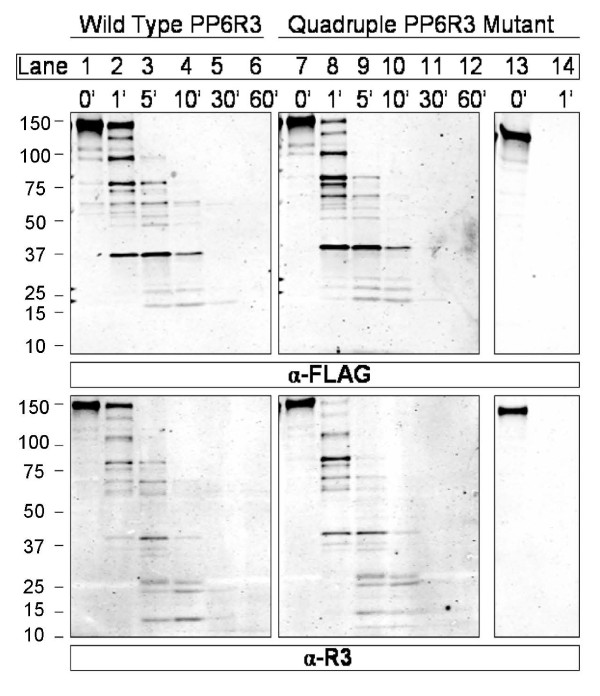
**Conformational integrity of quadruple mutated FLAG-PP6R3**. FLAG-tagged PP6R3 wild type (lanes 1-6) and the quadruple mutant E204K, E205K, E259K, E262K (lanes 7-14) were expressed in HEK293T cells, recovered by immunoprecipitation and digested with chymotrypsin for indicated periods of time. Aliquots of the reactions were resolved by SDS-PAGE and immunoblotted for both FLAG (upper panels) and PP6R3 (lower panels). As a control quadruple mutant protein eluted from beads (lane 13) was denatured and digested with the same amount of chymotrypsin for one min (lane 14) and analyzed by immunoblotting. Lines on left edge of panels show migration of molecular size standards.

Mutations that result in a loss-of-function always leave open the possibility that alteration of overall protein conformation causes the loss of function, rather than a local change of a particular side chain. This is even true for charged resides that are likely to appear on the protein surface. To compare the conformation of wild type and quadruple mutated (204/205/259/262) PP6R3 we utilized partial proteolysis by chymotrypsin (Figure 7). This protease cleaves C terminal to aromatic residues and therefore mutation of charged residues should not affect its reaction. Indeed, we observed essentially identical patterns of chymotrypsin digestion for wild type and the quadruple mutant FLAG-PP6R3. Both the kinetics of the reaction over the first 10 min and the sizes of fragments formed were indistinguishable. The fragments were reactive with both anti-FLAG (upper panels) and anti-PP6R3 antibodies (lower panels), indicating that the primary sites of proteolysis were toward the C terminal region of the protein. We noted that the N terminal region (~37 kDa) of PP6R3 was relatively less susceptible to chymotrypsin, because a FLAG-tagged fragment of this size persisted longer than other fragments formed within the first min of digestion (lanes 3, 4, and 9, 10). These patterns of fragmentation reflected conformational constraints on proteolysis because denaturation of the FLAG-PP6R3 resulted in its complete degradation in less than one min in a parallel reaction (lanes 13-14). We concluded that the mutation of the negative to positive charges in the predicted loops that severely reduced binding of PP6c did not cause a major change in conformation of PP6R3.

## Discussion

This study examined the properties of PP6R3, as an example of the conserved family of SAPS domain subunits that are specific for binding to PP6 phosphatase. The PP6R3 co-precipitated half the endogenous PP6c in different cell lines, showing it was the major binding partner for PP6, compared to the other SAPS subunits, and other proteins known to bind PP6c, such as alpha4 [22-24] and TIP [25], a.k.a. TAB4 [26]. Expression of the human SAPS subunits in yeast allowed for the co-precipitation of endogenous Sit4 with each of the SAPS. However, only PP6R3 fully rescued strains deleted for all yeast SAPS with restoration of budding, indicative of cell cycle progression [16]. We conclude that PP6R3 is a dominant and functionally conserved cellular partner for PP6c, making it a suitable representative of the family of SAPS proteins.

Our hypothesis is that the SAPS region of PP6R3 recognizes PP6c using alpha helical repeats that present residues in the inter-helical loops for subunit-subunit interaction. This follows in part from what is known about the PP2A complex with its scaffold A or PR65 subunit [6-8], but diverges in specific details. The N terminal 513 residues of PP6R3 are sufficient for stable association with PP6c, showing truncation of the C terminal 350+ residues of PP6R3 does not eliminate, or even diminish binding of PP6c. The 1-513 fragment of PP6R3 supports co-precipitation of PP6c, but a 1-355 fragment is not sufficient, suggesting that residues between 355 and 513 are critical, either for direct contact with the catalytic subunit, or for maintenance of the conformation of the SAPS domain. The inability of the 1-355 fragment to co-precipitate PP6c does not imply that it is not required or involved, just that it alone is insufficient. Clearly, point mutations within the 1-355 region (at 204, 205, 259, 262) were effective at nearly eliminating PP6c binding to full length PP6R3. This at least implies that these residues are somehow required and even dominant, though not alone sufficient, for stable subunit-subunit association. We speculate that there are contacts between PP6c and two regions of the SAPS domain, involving residues in the 200-265 region as well as residues in the 355-513 region. Multiple points of contact may well be necessary to achieve the specificity for PP6 vs. PP2A. Determination of the co-crystal structure will be needed to visualize the spatial organization of the SAPS domain and interactions with PP6c.

As a preliminary step in understanding the SAPS domain organization, and as a guide for mutagenesis we used sequence alignments and jury modelling methods to produce models of the SAPS region of PP6R3. The sequences of SAPS domains are sufficiently conserved so as to allow discovery of orthologs in species from yeast to mammals [14], and have been assigned a Pfam  designation, however the sequences do not allow a match to any structure based on available algorithms. The SAPS domain is predicted to have regions of alpha helical secondary structure, and the CD spectrum of an isolated SAPS domain indicates predominantly alpha-helical organization. We turned to jury modelling methods to produce hypothetical structures for the SAPS domain, using other known helical repeat proteins such as importins, beta-catenin, PP2A scaffolding PR65 subunit and golgin p115. These models resemble one another in that they are made of multiple helices, but each has different helix segments, inter-helix loops, and positioning of the helices relative to one another. Because previous studies of PP2A binding to its helical scaffolding (PR65) or regulatory subunit alpha4 showed basic residues on the phosphatase catalytic subunit were required, and these presumably paired with acidic side chains in other subunit [27], we focused on acidic residues in PP6R3 as likely sites for contacts with PP6c. Reduced PP6 binding due to charge reversal mutations was taken as evidence for involvement of specific residues in subunit-subunit association. This analysis implicated E204, E205, E2659 and E262 as possible participants. Helical repeat models for SAPS domain based on beta-catenin (Figure 5B), or on golgin p115 (Figure 5C) position residues 204, 205, 259 and 262 proximal to one another, on the same side of the protein surface, in contrast to the 3D model based on PR65 A subunit (Figure 5A) that positions these loops on opposite sides. Therefore, results of our mutagenesis studies suggest either model B or C in Figure 5 for arrangement of the alpha helices in the SAPS domain.

The sensitivity of PP6R3 to proteolytic cleavage about 330-350 residues from the N terminus was noted with trypsin digestion of purified recombinant GST fusion protein, and with endogenous protease cleavage of various sized FLAG-tagged proteins expressed in intact cells, and with chymotrypsin digestion of FLAG-tagged PP6R3 immunoprecipitated from cells. In the 3D model based on the golgin p115 structure (Figure 5C) residues 320-340 are not in a helix, but instead predicted to be in an exposed surface loop (Figure 4), whereas in the beta-catenin structural model (Figure 5B) this segment is mostly in a helical conformation. There are two tandem Pro-Pro sequences at 323-324 and 338-339 likely to prevent alpha helix formation and the intervening sequence contains KKS and TWG as possible sites for trypsin and chymotrypsin. This putative surface loop and the juxtaposition of the 204, 205, 259 and 262 residues makes the golgin p115 structure, compose of ARM repeats, our favored model for the SAPS domain in PP6R3. We speculate that some structural element in the 350-513 sequence region, following this loop in the golgin p115 structure, constitutes a required contact site for binding to PP6c. The low sequence similarity between the golgin p115 and other ARM repeat proteins, despite ARM structures that are remarkably similar, tells us that the sampling of ARM, HEAT, ANK and PUM repeat proteins in the PDB database is still limited. This may account for why the SAPS domain sequence did not match any known structures.

Finally, binding of the PP6 catalytic subunit to the SAPS region of PP6R3 leaves the C terminal region of PP6R3 for interaction with the third subunit of the PP6 trimer, one of the ankyrin-repeat subunits (ARS), proteins known previously as Ankrd28, Ankrd44 and Ankrd52. This hypothetical arrangement is similar to, but different from, the actual 3D structures determined for PP2A heterotrimers by X-ray crystallography. Most obvious, the entire A subunit for PP2A is HEAT repeats, while no predictions suggest the same is true for PP6 subunits, where the SAPS domain only includes the N terminal half of the protein. The PR65 or A subunit scaffold for PP2A is an open arc of side-by-side helices in the AC dimer, but bends into a more closed horseshoe or letter "C" shape in the ABC trimer structures (see Fig. 5A). The B regulatory subunits or proteins such as small t antigen evidently induce the conformational change in the scaffold subunit [28]. Other evidence prior to the determination of the crystal structure indicated that small t antigen affected the conformation of the PP2A scaffold subunit [29]. The protease sensitivity of PP6R3 at ~330 residues from N terminus suggests that there may be a flexible or disordered and exposed junction. In PP6 trimers the subunit made of ankyrin repeats is expected to contact PP6c to alter substrate specificity while primarily tethered to the C terminal region of PP6R3, which alone is sufficient for stable association. The structures of known ankyrin-repeat proteins such as ankyrin, IκB, and MYPT1 show that neighboring helical repeats produce curvature in the overall structure [1,3]. Thus, we imagine that the PP6 catalytic and ARS subunits could come into proximity to one another by their mutual binding to PP6R3. Such a model, like what is seen with PP2A, would predict that substrate specificity and possible regulation of activity arises from interaction of the catalytic and regulatory subunits (ARS) brought together by tethering to a common scaffold (SAPS subunit).

## Conclusion

In conclusion, the conserved SAPS domain in PP6R3 forms helical repeats that are probably organized similar to those in golgin p115. A core structure of about 350 N terminal residues is relatively protease-resistant and linked by a readily cleaved exposed segment to the rest of the SAPS region that is required for stable association with catalytic subunit. Charged residues in interhelical loops are used as primary contacts for specific recognition of PP6 and Sit4 catalytic subunits relative to other type-2A phosphatases.

## Authors' contributions

JG prepared antibodies and recombinant proteins, recorded the CD spectrum, produced the mutants, carried out pull-down assays and drafted portions of the manuscript, UD carried out sequence alignments and modelling, prepared Figures and drafted portions of the manuscript, JRE prepared recombinant proteins, carried out proteolysis analyses and drafted portions of the manuscript, DLB designed, organized and coordinated the studies, analyzed data, drafted portions of the manuscript and edited the final text. All authors read and approved the final manuscript.
